# Special Issue: Toxicity of Metals, Metal-Based Drugs, and Microplastics

**DOI:** 10.3390/ijms27146257

**Published:** 2026-07-14

**Authors:** Agnieszka Ścibior, Manuel Aureliano, Juan Llopis

**Affiliations:** 1 Laboratory of Oxidative Stress, Department of Biomedicine and Environmental Research, The John Paul II Catholic University of Lublin, 20-708 Lublin, Poland; 2 Faculdade de Ciências e Tecnologia (FCT), Campus de Gambelas, Universidade do Algarve, 8005-139 Faro, Portugal; 3 Centro de Ciências do Mar do Algarve (CCMAR/CIMAR LA), Campus de Gambelas, Universidade do Algarve, 8005-139 Faro, Portugal; 4 Department of Physiology, Institute of Nutrition and Food Technology, Biomedial Research Center, Institute of Biosanitary Research, University of Granada, 18071 Granada, Spain; jllopis@ugr.es

## 1. Introduction and Scope

**Figure 1:**
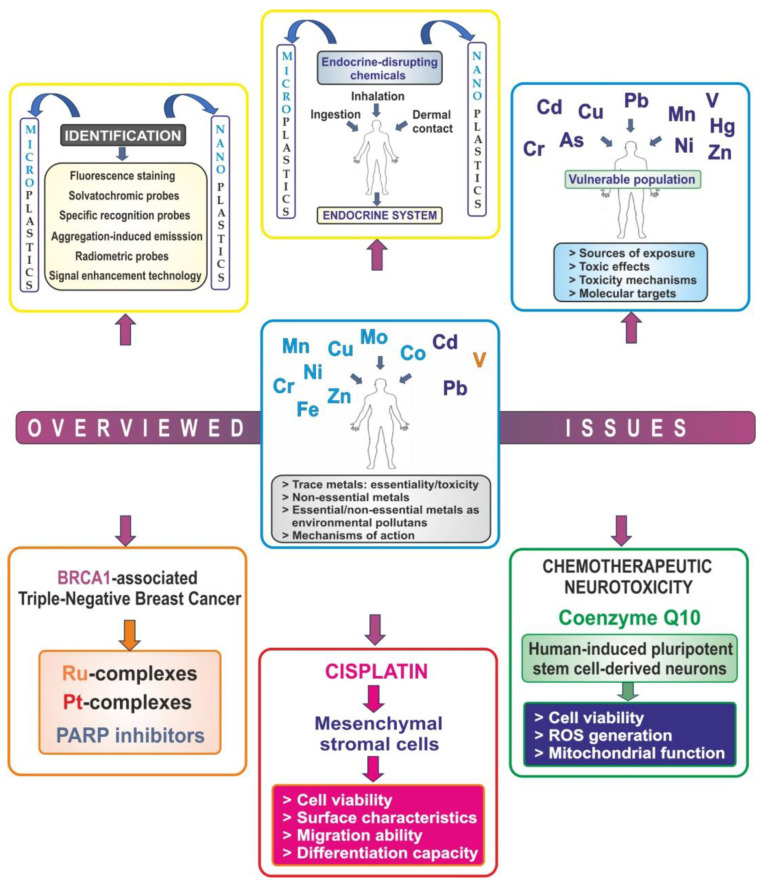
Graphical summary of included issues: a review on micro- and nanoplastics (yellow), a review involving metals (blue) and metal complexes (orange), studies on chemotherapeutic drugs (red), and studies with antioxidants (green).

In the present Special Issue (SI), titled “Toxicity of Metals, Metal-Based Drugs, and Microplastics”, an attempt has been made to include reports updating our knowledge about micro- and nanoplastics, certain airborne metals, some metals/metal complexes, chemotherapeutic drugs, and antioxidants. More precisely, this SI provides a review on the development of fluorescent probes essential for monitoring the presence of micro- and nanoplastics in the environment, examines micro- and nanoplastics as endocrine-disrupting factors, addresses the sources, toxicokinetics, and toxicodynamics of the most relevant metals associated with air pollution and their effects on vulnerable populations, describes some trace metals as essential nutrients and environmental toxicants, discusses the therapeutic potential of metal-based and PARP inhibitor chemotherapy for BRCA1-associated triple-negative breast cancer, and collects data on the influence of cisplatin on the functionality and surface characteristics of mesenchymal stromal cells. In addition, this SI provides valuable findings on the importance of some natural antioxidants like Coenzyme Q10 in addressing the neurotoxic impacts of chemotherapy. The scientific articles making up this SI, i.e., five review articles and two original papers (seven in total), have garnered a total of 44 citations and 23,143 views, indicating an average of 6 citations and 3306 views per publication (26 June 2026). Figure 1 summarizes the issues included in this SI.

## 2. Contributions

The contributions are organized in four subsections: a review on micro- and nanoplastics (two articles), a review involving metals (two articles) and metal complexes (one article), studies on chemotherapeutic drugs (one article), and studies with antioxidants (one article) (Figure 1).

### 2.1. Micro- and Nanoplastics

The first paper by Yang et al. [contribution 1] published in this Special Issue, titled “Illuminating the invisible: Fluorescent probes as emerging tools for micro/nanoplastic identification”, provides a comprehensive overview on the development and application of detection techniques for identifying micro- and nanoplastics (MNPs), which have become ubiquitous in the environment, raising concerns about their potential harm to humans. It summarizes advanced strategies that can overcome the limitations of traditional methods, which are characterized by low sensitivity and efficiency. The article discusses detection techniques from basic hydrophobic adsorption staining methods such as Nile Red and Coumarin 6 to modern strategies such as solvatochromic and aggregation-induced emission (AIE) probes and signal amplification technologies (Plasmon-enhanced fluorescence), which improve detection sensitivity. It should be stressed that the incorporation of innovative strategies enhances detection capabilities at very low concentrations. In the context of practical applications, the fluorescent method is described as a fast and effective technique facilitating accurate detection of MNPs, which is crucial for monitoring their presence in the environment and taking environmental protection measures.

Another review article, titled “Micro- and nanoplastics as disruptors of the endocrine system—A review of the threats and consequences associated with plastic exposure”, included in this SI [contribution 2], summarizes the current state of knowledge about the effects of micro- and nanoplastics (MNPs) containing endocrine-disrupting chemicals (EDCs) on the endocrine system. The paper is supplemented with three brief summary tables which collect information about the main types of plastics found in the environment, their typical uses, and degradation characteristics as well as about the effects of MNPs on the ovaries and testes. It also contains two figures which illustrate the routes and sources of exposure of humans to MNPs as well as hormonal axes with their main hormones. The authors explore the toxicological impact of MNPs on individual endocrine glands such as hypothalamus, pituitary gland, pineal body, thyroid gland, parathyroids, adrenal glands, ovaries, and testes as well as on key endocrinological regulatory axes, including hypothalamic–pituitary–gonadal, hypothalamic–pituitary–thyroid, and hypothalamic–pituitary–adrenal axes. The data provided in this review clearly indicate that MNPs disrupt various endocrine organs and dysregulate hormonal axes. This work has a positive impact on education, as the knowledge about the potential health effects associated with the exposure to MNPs may lead readers to take preventive actions and raise their environmental awareness, prompting reflection on their own contribution to environmental pollution. The authors of this review not only draw attention to the need to limit the mass production of plastics, especially the production of EDC additives, but also highlight the necessity of further research to examine the long-term effects of the exposure to MNPs and to elucidate the molecular and epigenetic mechanisms of their action. Moreover, they point to the need to identify early biomarkers of toxicity and to conduct detailed epidemiological studies to establish links between the exposure to MNPs and health outcomes.

### 2.2. Metals and Metal Complexes

One of the review articles, titled “Metal pollution in the air and its effects on vulnerable populations: A narrative review” published in this SI [contribution 3], examines the effects of selected metals, both essential and/or highly toxic, such as arsenic (As), cadmium (Cd), chromium (Cr), copper (Cu), lead (Pb), mercury (Hg), manganese (Mn), nickel (Ni), vanadium (V), and zinc (Zn), on vulnerable populations, including pregnant women, children, adolescents, older adults, occupationally exposed workers, and individuals with metabolic diseases, who are at increased risk of development of health problems. The manuscript contains brief summary tables in which the authors collect data on the toxic effects of all the above-mentioned metals present in air pollution on each vulnerable population and on the molecular targets and toxicity mechanisms of each of these elements, which enhances the review’s usefulness for readers. This review, which is scientifically significant and important from the public health perspective, clearly shows that the exposure to metals is a serious environmental and public health problem. Based on the data presented in this work, it can be concluded that prevention of metal pollution is critical for protecting health, particularly in vulnerable populations. There is also a need for introducing proactive measures to reduce exposure and health risks associated with metal contamination. It should also be mentioned that the widespread impact of metals on human health highlights the necessity of a comprehensive analysis of their toxicity mechanisms. Therefore, toxicological research on various metals that can interact within the organism is very important and indispensable. The authors of this review point to the need for further studies which, as they stress, should include other susceptibilities across different populations and analyze disaggregated data based on, among others, age, sex, dose, duration of exposure, and metabolic and chronic diseases, which will ensure a better understanding of the effects of metals on vulnerable populations.

Another review article, titled “The dual nature to metals: Essential nutrients and environmental contaminants”, included in this SI [contribution 4], focuses on the essentiality and toxicity of such trace elements as iron (Fe), zinc (Zn), copper (Cu), manganese (Mn), chromium (Cr), molybdenum (Mo), and nickel (Ni), as well as on the toxicity of non-essential metals for humans, such as lead (Pb) and cadmium (Cd), and on vanadium (V), whose essentiality for humans is actually not well defined and is a subject of discussion. It also describes the main mechanisms of action by which the above-mentioned metals exert their toxic effects and the main natural and anthropogenic sources of emissions of essential and non-essential metals into the environment.

In turn, the review paper titled “Therapeutic potential of metal-based and PARP inhibitor chemotherapy for BRCA1-associated triple-negative breast cancer” included in this SI [contribution 5] comprehensively explores the therapeutic potential of metal-based drugs, such as platinum (Pt) and ruthenium (Ru) compounds, and DNA-damaging agents, such as poly (ADP-ribose) polymerase (PARP) inhibitors, in treating BRCA1-mutated triple-negative breast cancer (TNBC), which is the most aggressive form of the disease [1]. BRCA1-associated TNBC is characterized by rapid growth and a higher risk of recurrence, which makes traditional therapies potentially less effective. Generally, patients with TNBC have a less favorable prognosis and survival rate than those with other types of breast cancer. They often respond less well to conventional chemotherapy, compared to other groups of breast cancer patients [1,2]. Therefore, this type of breast cancer requires innovative, personalized therapeutic approaches to improve treatment outcomes and patient quality of life. The tables included in the review, which summarize the mechanisms, toxicity, and efficacy of Pt-complexes, Ru-complexes, and PARP inhibitors on TNBC and compare the mechanisms, advantages, limitations, and clinical status of Pt drugs, Ru-complexes, and PARP inhibitors, clarify the knowledge, rendering the text clearer for the reader. The authors of this work emphasize the clinical challenges of TNBC related to the lack of targeted therapies, address cellular resistance to Pt drugs and PARP inhibitors, and draw attention to the need for a better understanding of the precise mechanisms of action of the anticancer metal-based candidates and their potential synergy with existing therapeutic drugs. Such an approach, as they stress, can pave the way for the development of novel metal-based drugs which will be able to overcome treatment resistance and improve efficacy and selectivity. The integration of metal-based chemotherapy and PARP inhibitors can represent a promising advancement in the treatment of BRCA-1-associated triple-negative breast cancer, offering hope for improving patient outcomes.

### 2.3. Chemotherapeutic Drugs

One of the original articles, titled “The influence of cisplatin on functionality and surface characteristics of mesenchymal stromal cells in vitro” written by von Fournier and co-workers, included in this SI [contribution 6], focuses on the in vitro influence of cisplatin on the functionality and surface characteristics of mesenchymal stromal cells (MSCs), which play a crucial role in regenerative medicine due to their multipotent differentiation capacity [3] and influence on tumor biology [4]. The aim of this study was to evaluate the impact of different doses of cisplatin on MSCs from four human donors on their viability, surface markers, and capability of differentiation and migration. As far as the cytotoxicity of cisplatin (0.1 µM, 0.5 µM, 1 µM, 2 µM, 5 µM, 10 µM, 20 µM, and 100 µM) on MSCs is concerned, a significant reduction in viability was detected at 100 µM; in turn, at 10 µM and 20 µM, only a reduction trend in viability was observed (MTT assay). As for the surface markers, such surface antigens as CD73, CD90, and CD105 were detected, whereas CD31, CD34, and CD45 were not detected on the surface of the cells under the treatment with 2 µM or 10 µM of cisplatin (flow cytometry). To investigate the influence of cisplatin (2 µM, 10 µM, and 2 µM for 5 days) on differentiation (osteogenic, chondrogenic, and adipogenic), the expression of leptin (adipogenic differentiation), SOX9 (chondrogenic differentiation), and RunX (osteogenic differentiation) was measured by the RT-qPCR technique in one donor. The expression of leptin and SOX9 was observed to be decreased, whereas the RunX expression was increased. In the second donor, the expression of leptin was also reduced after the cisplatin (10 µM) exposure, but the expression of SOX9 was elevated after the treatment with 10 µM of cisplatin and decreased after the prolonged treatment (i.e., after 5 days) with 10 µM of this chemotherapeutic drug. Thus, diverge results were noted for chondrogenic differentiation (both donors). The authors hypothesized an inhibitory effect of cisplatin on adipogenic differentiation and a rather enhancing effect on osteogenic and chondrogenic differentiation. The histological analysis showed no difference with regard to osteogenic, chondrogenic, and adipogenic differentiation at doses up to 10 µM. Cell migration was not restricted by the cisplatin exposure (scratch assay). Based on the obtained findings, the authors highlighted that larger multi-donor cohorts with extended cisplatin dosing are needed to establish robustness and clinical relevance and to determine whether the observed changes translate into functional effects on mesenchymal stromal cells differentiation. The above-mentioned study is an interesting topic in the field of cancer therapy, as it provides important information about the impact of cisplatin on MSCs, which may be significant for future therapeutic strategies in cancer treatment. However, there is a need for more extensive studies on the interactions between cisplatin and MSCs in order to better understand their role in oncological therapies and their impact on tissue regeneration after chemotherapy.

### 2.4. Antioxidants

Another original article, titled “Coenzyme Q10 ameliorates chemotherapy-induced neurotoxicity in iPSC-derived neurons by reducing oxidative stress”, included in this SI [contribution 7], explores the efficacy of Coenzyme Q10 (CoQ10) in counteracting the effects of five chemotherapeutic drugs, i.e., 5-fluorouracil, methotrexate, and paclitaxel (0, 1, and 10 µM), as well as doxorubicin and vincristine (0, 0.1, and 1 µM) on cell viability, ROS levels, and mitochondrial membrane potential using a model of human induced pluripotent stem cell (iPSC)-derived neurons. The authors showed that CoQ10 (10 µM) improved neuronal viability following the exposure of iPSC-derived motor neuron progenitors (iPSC-MNPs) to all five chemotherapeutic agents. Moreover, they demonstrated that the treatment of human iPSC-derived neurons with CoQ10 under chemotherapeutic stress resulted in a reduction in the intracellular production of ROS, which are responsible for neuronal damage associated with chemotherapy [5], and elevation of mitochondrial function, which is crucial for cell survival and health [6], in a drug- and dose-dependent manner. It should be highlighted that neurotoxicity is a common and potentially serious adverse effect of both conventional and novel cancer therapy [7] and that chemotherapy-induced neurotoxicity may affect any neuron in the body and cause many different symptoms affecting the quality of life of patients undergoing anti-cancer treatment [8]. Therefore, this study, which was designed to focus on the neuroprotective potential of CoQ10 against chemotherapy-induced neurotoxicity by using iPSC-MNPs, is very important, as it offers insight into the potential therapeutic use of this naturally occurring antioxidant. The authors of this work emphasize the need for further research to validate the obtained findings which may lay the groundwork for future preclinical studies aimed at reducing the neurological burden of cancer therapy.

## 3. Conclusions and Outlook

This SI provides valuable information on the analytical methods for detecting micro- and nanoplastics, which have become ubiquitous environmental pollutants and pose a significant threat to both the environment and public health. In other words, it provides the reader with innovative detection tools that are crucial for monitoring the problem of pollution caused by micro- and nanoplastics. It should be highlighted that the problem of pollution with plastic, which is used in almost all consumer and industrial activities [9], is one of the most important environmental challenges of the modern world.

This SI also summarizes the current state of knowledge about the endocrine-disrupting effects of micro- and nanoplastics (MNPs). Recent studies have confirmed the presence of microplastics in human tissues, raising serious concerns about their potential toxicological effects [10]. As MNPs can pose a serious threat to human health due to their ability to disrupt hormonal functions, with potential long-term consequences for future generations, it is important to find effective ways to mitigate the exposure to these compounds in order to reduce the associated health risks. Based on the findings included in this review focusing on the impact of MNPs on the endocrinological system, it can be concluded that there is a need for interdisciplinary research to clarify the exact mechanisms of their toxicity and develop regulatory frameworks aimed at reducing the human health risks associated with plastics.

This SI also comprehensively discusses how some populations are more vulnerable to the effects of airborne metal pollution and helps in identifying groups that require special attention in public health protection. The information contained in this review may serve as a basis for strategies aimed at reducing metal emissions and implementing actions to protect public health, especially in areas with the highest metal exposure. In addition, this work raises social awareness about the problem of air pollution and can stimulate actions both at the individual and governmental levels. From this SI, the reader also gains knowledge about the impact of essential and non-essential elements for humans, as well as awareness of environmental hazards resulting from human activity.

Moreover, this SI addresses the therapeutic potential of metal-based and PARP inhibitor chemotherapy for BRCA1-mutated triple-negative breast cancer, which, as the authors stressed, accounts for about 10–15% of all breast cancers. As known, this type of cancer is the leading cause of cancer-related mortality among females and is a leading global health challenge [11]. It should be noted that the data presented in this review point to the need to continue research on breast cancer and to develop more effective therapies for patients who lack effective treatment options. The use of modern therapies can lead to a more personalized approach in oncology, where treatment is tailored to the specific genetic characteristics of the patient. It should also not be ruled out that the information contained in this review may impact future clinical trials and inspire researchers to examine other types of cancer as well as lead to greater awareness among researchers and physicians, which may consequently contribute to progress in breast cancer research.

Furthermore, this SI collects data on the influence of cisplatin on mesenchymal stromal cells (MSCs) with regard to their defining characteristics and their ability to differentiate and migrate, which may have significance for future therapeutic strategies in cancer treatment. The elucidation of the effect of cisplatin on MSCs may lead to the development of therapeutic strategies that minimize the negative effects of chemotherapy, which will increase the effectiveness of cancer treatment and support tissue regeneration.

Finally, this SI also contains findings which suggest that CoQ10 could be a valuable therapeutic strategy to mitigate neurological side effects in patients undergoing chemotherapy. This completely innovative approach for alleviating chemotherapy effects may have clinical significance for oncological patients. Studying the effectiveness of CoQ10 as a means of alleviating neurotoxicity opens new avenues, i.e., it offers a potential solution for patients experiencing side effects from cancer treatment. The results presented in this work may motivate further research on supplementation with CoQ10 and its effects on other aspects of health, especially in the context of cancer therapy, and lead to future development of clinical recommendations regarding the use of CoQ10 in supportive therapy in patients undergoing chemotherapy, which may contribute to improving their quality of life.

We believe that the information provided in this SI will not only stimulate readership interest in the field of micro- and nanoplastics in general but also inspire researchers engaged in the field of metals and metal complexes. We also think that the present SI will be relevant to those interested in natural antioxidants and to those focused on chemotherapeutic drugs.
